# Alzheimer’s disease like neuropathology in Down syndrome cortical organoids

**DOI:** 10.3389/fncel.2022.1050432

**Published:** 2022-12-08

**Authors:** Helen H. Zhao, Gabriel G. Haddad

**Affiliations:** ^1^Department of Pediatrics, University of California San Diego, La Jolla, CA, United States; ^2^Department of Neurosciences, University of California San Diego, La Jolla, CA, United States; ^3^The Rady Children’s Hospital, San Diego, CA, United States

**Keywords:** Down syndrome, Alzheimer’s disease (AD), iPSC, proliferation, amyloid-beta, tau pathology, cortical organoid

## Abstract

**Introduction:** Down syndrome (DS) is a genetic disorder with an extra copy of chromosome 21 and DS remains one of the most common causes of intellectual disabilities in humans. All DS patients have Alzheimer’s disease (AD)-like neuropathological changes including accumulation of plaques and tangles by their 40s, much earlier than the onset of such neuropathological changes in AD patients. Due to the lack of human samples and appropriate techniques, our understanding of DS neuropathology during brain development or before the clinical onset of the disease remains largely unexplored at the cellular and molecular levels.

**Methods:** We used induced pluripotent stem cell (iPSC) and iPSC-derived 3D cortical organoids to model Alzheimer’s disease in Down syndrome and explore the earliest cellular and molecular changes during DS fetal brain development.

**Results:** We report that DS iPSCs have a decreased growth rate than control iPSCs due to a decreased cell proliferation. DS iPSC-derived cortical organoids have a much higher immunoreactivity of amyloid beta (Aß) antibodies and a significantly higher amount of amyloid plaques than control organoids. Although Elisa results did not detect a difference of Aß40 and Aß42 level between the two groups, the ratio of Aß42/Aß40 in the detergent-insoluble fraction of DS organoids was significantly higher than control organoids. Furthermore, an increased Tau phosphorylation (pTau S396) in DS organoids was confirmed by immunostaining and Western blot. Elisa data demonstrated that the ratio of insoluble Tau/total Tau in DS organoids was significantly higher than control organoids.

**Conclusion:** DS iPSC-derived cortical organoids mimic AD-like pathophysiologyical phenotype *in vitro*, including abnormal Aß and insoluble Tau accumulation. The molecular neuropathologic signature of AD is present in DS much earlier than predicted, even in early fetal brain development, illustrating the notion that brain organoids maybe a good model to study early neurodegenerative conditions.

## Introduction

Down syndrome (DS) is a genetic disorder with an extra copy of chromosome 21, characterized by physical growth delay, mild to moderate intellectual disability, and characteristic facial features. DS is one of the most common causes of intellectual disabilities in humans, with an incidence of one in 700 newborns. Moreover, DS patients often have an increased risk to develop many other health problems including Alzheimer’s disease (AD), obstructive sleep apnea, congenital heart defect, and leukemia (Asim et al., 2015). Almost all DS patients have AD-like neuropathological changes (AD-DS) such as accumulation of plaques and tangles at about 40 years of age. Approximately 40%–80% of DS patients develop AD-like dementia by 50–60 years, much earlier than the majority of AD patients (Oliver and Holland, 1986; Holland et al., 1998; Zigman et al., 2004). DS and AD patients share many neuropathological changes such as amyloid beta accumulation, tau pathology, endosomal dysfunction, synaptic dysfunction, and neurogenesis defects. The earliest neuropathological changes such as enlarged endosomes, impaired synaptogenesis, and neurogenesis can be traced back to early life even at the fetal brain in DS patients (Marin-Padilla, 1972; Wisniewski et al., 1984; Cataldo et al., 2000; Baburamani et al., 2019; Patkee et al., 2020; Tang et al., 2021). However, due to the lack of human samples and appropriate techniques, our understanding of DS neuropathology during brain development or before the clinical onset of the disease remains largely unexplored at the cellular and molecular levels.

Induced pluripotent stem cell (iPSC) technology, first introduced by Yamanaka in 2007 (Takahashi et al., 2007) has been widely used by many laboratories to study a variety of human diseases, including Alzheimer’s disease (Kondo et al., 2013), Parkinson’s disease (Hargus et al., 2010), and Autism spectrum disorder (Russo et al., 2019). iPSC-derived 3D cortical organoids have been shown to closely simulate the key endogenous neurodevelopmental events with a cytoarchitecture resembling regions of the developing human brain (Paşca, 2018) and recapitulate the trajectory of human brain development and maturation (Lancaster and Knoblich, 2014; Paşca et al., 2015). Moreover, brain organoids have a transcriptome profile that is close to that in the early human brain (Camp et al., 2015; Paşca et al., 2015; Nascimento et al., 2019; Trujillo et al., 2019). Therefore, iPSC-derived brain organoids represent an optimized approach for modeling neurodevelopmental disorders such as Down syndrome and allow us to explore the earliest cellular and molecular changes during DS fetal brain development.

To our knowledge, currently only one published study used iPSC-derived organoids to investigate the AD-like pathology to compare a DS patient and a healthy control (Gonzalez et al., 2018). Here, by comparing DS-specific iPSC lines and their isogenic control iPSC lines, we demonstrate that abnormalities in Down syndrome start as early as the iPSC stage and AD-like neuropathological phenotype including abnormal Aß accumulation and Tau pathology progressively manifest themselves in organoids during early development.

## Materials and Methods

### Karyotype of iPSC lines

All iPSC lines were obtained from Dr. Stuart Orkin at Boston Children’s Hospital through a material transfer agreement. The use of cells was approved by IRB at the University of California San Diego. Two DS iPSC subclones and two isogenic control subclones were isolated from DS1-iPS4 cells as previously described (Maclean et al., 2012). The iPSCs were fed daily with mTeSR medium. DNA was extracted from iPSCs using a DNase Blood and Tissue kit (Qiagen, CA), the presence of an extra copy of chromosome 21 in DS iPSCs was confirmed by a high-resolution karyotyping performed by the Cell Line Genetics (Madison, WI).

### Real time PCR

RNA from iPSCs was extracted using the RNase Mini kit (Qiagen, CA). One microgram of total RNA was converted to complementary DNA using SuperScript First-Strand Synthesis System for RT-PCR (Life Technologies, CA). Real time PCR was performed using a GeneAmp 7900 sequence detection system with POWER SYBR Green (Applied Biosystems, CA). Gene expression of *GAPDH* levels was used as a loading control. Real time PCR data were presented after normalization with *GAPDH* expression. Primers used in the current study are listed in Table 1.

**Table 1:** List of primers.

**Genes**	**Primer sequence**
APP	FW: TTTGGCACTGCTCCTGCT
	RV: CCACAGAACATGGCAATCTG
DYRK1A	FW: CTGGACTCTTCCCTCCCTTC
	RV: GCCGAACAGATGAAGGTTTG
BACE2	FW: TGCCTGGGATTAAATGGAATGG
	RV: CAGGGAGTCGAAGAAGGTCTC
RCAN1	FW: GCGTGGTGGTCCATGTATGT
	RV: TGAGGTGGATCGGCGTGTA
CSTB	FW: ATTCAAGAGCCAGGTGGTCG
	RV: CACTCGCAGGTGTACGAAGT
DSCAM	FW: CCTACGAACACGCCAAGATG
	RV: TACTCATTTGTCCCTGCCGT
GAPDH	FW: GCACCGTCAAGGCTGAGAAC
	RV: CGCCCCACTTGATTTTGG

### Cell growth analysis

Both DS and isogenic control iPSCs were seeded in a 6-well plate with a density of 10^5^ live cells/well and total cells are collected and counted after 3, 5, and 7 days in culture using Bio-Rad TC20 cell counter. The cell numbers were compared between the two groups.

### Cell proliferation experiments

DS and isogenic control iPSCs were seeded in a 4-well chamber slide with a density of 10^4^ live cells/well and cultured for 5 days. Immunostaining of Ki67 in iPSCs was followed the standard immunofluorescence staining protocol. Double labeling of EdU and BrdU was performed as previously described (Deshpande et al., 2017) with minor modifications. In brief, iPSCs were treated with EdU (20 μM) for 1 h and then with BrdU (10 μM) for an additional 1 h of incubation. Cells were fixed with 4% paraformaldehyde for 15 min and then permeabilized with 0.5% triton in PBS for 20 min. DNA was denatured with 4 M HCl for 20 min and followed by phosphate citric acid buffer for 10 min. EdU detection was performed using Click-iT^TM^ Plus EdU Cell Proliferation Kit following the manufacturer’s protocol. BrdU detection was performed following the standard immunofluorescence protocol with BrdU antibody (clone MoBU-1). Nuclei were stained with Hoechst 33342. EdU^+^ and BrdU^+^ cells were compared between the two groups.

### Generation of cortical organoids

Cortical organoids were generated from iPSCs and organoid spheres were kept in suspension under rotation (95 rpm) as previously described (Camp et al., 2015; Trujillo et al., 2019; Yao et al., 2020). In brief, on day 0, iPSCs colonies were dissociated into single cells using accutase with PBS at a ratio of 1:1, approximately 4 × 10^6^ cells were transferred to one well of a 6-well plate in mTeSR1 supplemented with 5 μM Y-27632, 10 μM SB431542 (SB), and 1 μM Dorsomorphin (Dorso) for 3 days. Y-27632 was removed after 24 h; on day 3, mTeSR1 was substituted by base medium containing neurobasal, glutamax, 1% MEM nonessential amino acids (NEAA), 2% Gem21, and 1% penicillin/streptomycin (PS) supplemented with 1% N_2_, 10 μM SB, and 1 μM Dorso. The medium was changed every other day. On day 9, organoids were fed with a base medium supplemented with 20 ng/ml FGF2 for a week and the medium was changed every day. On day 16, the medium was switched to base medium supplemented with 20 ng/ml of FGF2 and 20 ng/ml EGF. On day 22, organoids were fed with the base medium supplemented with 10 ng/ml of BDNF, 10 ng/ml of GDNF, 10 ng/ml of NT-3, 200 μM L-ascorbic acid, and 1 mM dibutyryl-cAMP. Four weeks later, cortical organoids were maintained in the base medium with media changes twice a week. All reagents and chemicals used in the current study are listed in Table 2.

**Table 2:** List of reagents and chemicals.

**Reagents and kits**	**Catalog number**	**Company**
mTeSR1	85850	Stemcell technologies
Accutase	A6964	Sigma-Aldrich
Y-27632	125410	Fisher
SB431542	04-0010-10	Stemgent
Dorsomorphin	3093	Fisher
Neurobasal	21103049	Life Technologies
Glutamax	35050061	Life Technologies
MEM nonessential amino acids	11140050	Life Technologies
Gem21	400160	Gemini Bio-Products
penicillin/streptomycin	15140122	Life Technologies
N2 NeuroPlex	400163	Gemini Bio-Products
FGF2	PHG0263	Stemcell technologies
EGF	AF-100-15	PeproTech
BDNF	450-02	PeproTech
GDNF	450-10	PeproTech
NT3	450-03	PeproTech
L-ascorbic acid	A4403	Sigma-Aldrich
dibutyryl-cAMP	D0627	Sigma-Aldrich
BrdU (5-bromodeoxyuridin)	B23151	Themo fisher
Goat Anti-Rabbit IgG H&L (Alexa Fluor^®^ 555)	ab150078	Abcam
Goat Anti-Mouse IgG H&L (Alexa Fluor^®^ 488)	ab150113	Abcam
TRA-1-60 Antibody	SC-21705	Santa Cruz
Nanog (1E6C4) Mouse mAb	4893	Cell signaling
Nestin (E4O9E) XP^®^ Rabbit mAb	73349	Cell signaling
Anti-Sox-2 Antibody, clone 1A2	ZRB5603	Sigma
Anti-MAP2 antibody	Ab5392	Abcam
S100 Beta Polyclonal antibody rabbit	15146-1-AP	Proteintech
anti-cleaved-caspase-3 (rabbit)	9661S	Cell signalling
anti-Ki67 (rabbit)	ab15580	Abcam
BrdU Monoclonal Antibody (MoBU-1)	B35141	Thermofisher
β-Amyloid (D54D2) XP^®^ Rabbit	8243S	Cell signaling
Amyloid beta (N) (82E1) anti-human mouse IGG	10323	IBL-America
Amylo-Glo RTD Amyloid Plaque Stain Reagent	TR-300-AG	Biosensis
Phospho-Tau (Ser396) Polyclonal Antibody	44-752G	Thermofisher
Tau Antibody (A-10)	SC-390476	Santa Cruz
GAPDH antibody	2118s	Cell signaling
guanidine-HCl	G3272	Sigma
RIPA buffer	9806S	Cell signaling
protease inhibitor	1860932	Thermoscientific
PMSF	8553S	Cell signaling
Click-iT^TM^ Plus EdU Cell Proliferation Kit for Imaging, Alexa Fluor^TM^ 594 dye	C10639	Themofisher
Tau (Total) Human ELISA Kit	KHB0041	Themofisher
Amyloid beta 40 Human ELISA Kit	KHB3481	Life Technologies
Amyloid beta 42 Human ELISA Kit	KHB3441	Life Technologies

### Elisa of amyloid beta and tau

Detergent soluble and insoluble fractions of amyloid beta (Aβ40, Aβ42) and total Tau were quantified using Elisa kits following the manufacturer’s instructions. Soluble and insoluble fractions were prepared based on the previously published study (Wang et al., 2021). In brief, organoids were homogenized in RIPA buffer with protease inhibitors, the homogenate was centrifuged at 15,000× *g* for 10 min, and the supernatant was collected to yield a detergent soluble fraction. The remaining organoid pellet was then resuspended in 5 M guanidine-HCl diluted in 50 mM Tris, pH 8.0 with protease inhibitor and mechanically agitated at room temperature for 4 h to extract the detergent-insoluble fraction. Guanidine treated samples were diluted 1:2 in sterile PBS and centrifuged at 16,000× *g* for 20 min. The supernatant was collected to yield an insoluble fraction. The protein concentration of both soluble and insoluble supernatants was determined using a BCA assay and an equal amount of total protein was used for the Elisa assay.

### Immunofluorescence staining

Organoids were fixed with 4% paraformaldehyde for 30 min and then were transferred to 30% sucrose solution at 4°C overnight. Cryopreserved organoids were embedded in OCT and sectioned into 14 μm-thick slices for immunofluorescence staining. The sections were treated with 0.5% triton in PBS for 20 min and followed by blocking with 5% BSA in PBS for 1 h at room temperature. The sections were then incubated with the primary antibody (see Table 2) diluted in PBS with 5% BSA overnight and Alexa 488 and Alexa 555 conjugated secondary antibodies to specific IgG types for 1 h. Prolong Diamond anti-fade mountant with DAPI was used as a counterstain (Sigma, St. Louis, MO).

### Amylo-Glo staining

Amyloid plaque staining on organoid sections was performed using the Amylo-Glo RTD amyloid plaque stain reagent (Biosensis, Australia) following the manufacturer’s instructions.

### Western blot

Twenty micrograms of soluble protein from 12 weeks DS and control organoids were separated on NuPAGE 4%–12% Bis-Tris gels and then transferred to polyvinylidene difluoride membranes (Millipore, CA). The membranes were probed with a primary antibody in PBST with 5% BSA overnight at 4°C and followed by an appropriate horseradish peroxidase-conjugated secondary antibody (Invitrogen, CA). Immunoreactive bands were visualized using Bio-Rad ChemiDoc XRS with enhanced chemiluminescence (Perkin Elmer, MA). Equal loading was assessed using GAPDH and data were analyzed using ImageLab software (version 3.0, Bio-Rad).

#### Imaging analysis

All immunofluorescence images were captured using a 20× objective on a Nikon A1 confocal microscopy with NIS elements AR 5.20.02 software (Nikon Instruments Inc, Melville, NY). All images that were compared were obtained with identical settings and quantified using FIJI/ImageJ (version 2.5.0).

#### Statistical analyses

All data analysis and plots were done by OriginPro 2018b software (Northampton, MA, USA). All data were subjected to Shapiro-Wilk normality testing, normally distributed data were analyzed by one way ANOVA and non-normal distributed data were analyzed by non-parametric Mann-Whitney test. Results are expressed as mean ± SEM and the threshold for statistical significance (*p*-value) was set at 0.05. All the experiments were repeated at least three times.

## Results

### Characterization of DS and isogenic control iPSCs

**Figure 1:**
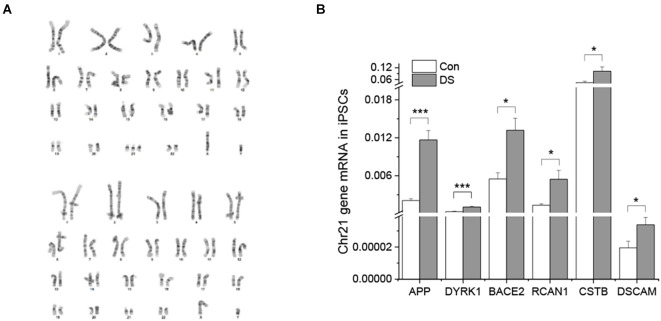
Characterizations of DS iPSC and isogenic control cell lines. **(A)** DS iPSC (top) and isogenic control (bottom) lines have correct karyotypes. **(B)** Overexpression of chromosome 21 genes in DS iPSCs was confirmed by real time PCR (*n* = 4). *p < 0.05 and ***p < 0.001 vs. control.

To confirm the presence of an extra copy of chromosome 21 in DS lines, stem array, a higher resolution of karyotyping, was performed and confirmed that DS iPSC lines indeed have a third copy of chromosome 21 while the isogenic control iPSC lines have a normal karyotype (Figure 1A).

To examine whether an extra copy of chromosome 21 results in overexpression of chromosome 21-encoded genes, we compared the expression profile of six genes between DS iPSCs and isogenic control iPSCs using real time PCR. Five of them were known to be overexpressed in both DS and AD patients (Gomez et al., 2020) and DSCAM was known to play an important role in synaptic plasticity and maturation (Stachowicz, 2018; Chen et al., 2022). As shown in Figure 1B, all six chromosome 21-encoded genes tested have a significantly increased expression in DS iPSCs as compared to isogenic controls (Figure 1B and Supplementary Figure 1, *p* < 0.05 or *p* < 0.001), indicating a gene dose effect of trisomy 21 in DS lines.

### Decreased cell growth in DS iPSCs

**Figure 2:**
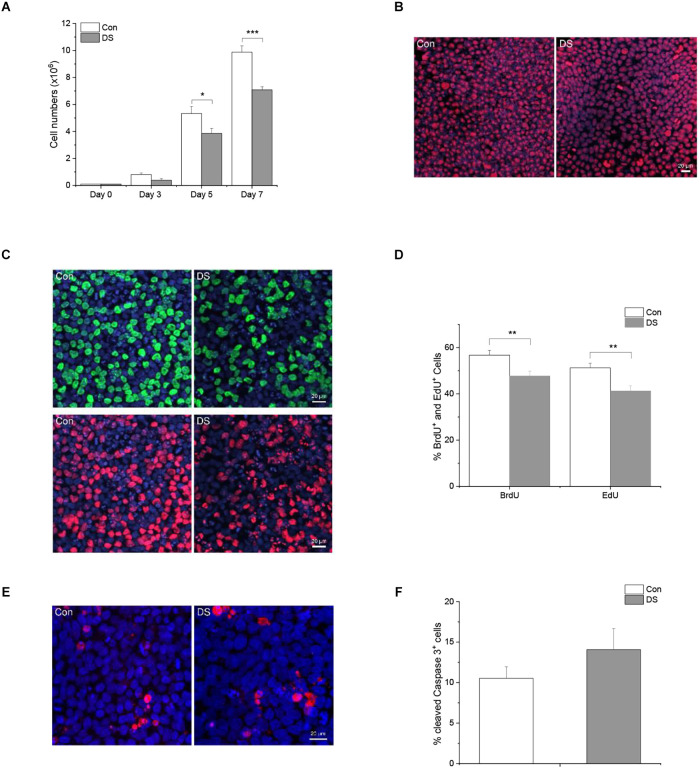
Decreased cell growth in DS iPS cells. **(A)** iPSCs were seeded in a 6-well plate with a density of 10^5^ live cells/well and total cell numbers were counted after 3, 5, and 7 days in culture. DS iPSCs have a significantly reduced cell number as compared to control iPSCs at day 5 (*n* = 12, *p* < 0.05) and day 7 (*n* = 12, *p* < 0.001). **(B)** A representative immunostaining of Ki67 in DS and control iPSCs. **(C)** A representative double labeling of BrdU (green) and EdU (red) in DS and control iPSCs. **(D)** Immunohistochemical analysis of BrdU^+^ and EdU^+^ cells revealed that DS iPSCs have a significantly decreased number of BrdU^+^ and EdU^+^ cells than control iPSCs (*n* = 1,690–1,897 cells, *p* < 0.01). **(E)** A representative immunostaining of cleaved caspase 3 in DS and control iPSCs. **(F)** Immunohistochemical analysis of cleaved caspase 3 revealed a similar percentage of cell death in DS and control iPSCs (*n* = 1,045–1,570 cells, *p* > 0.05). *p < 0.05, **p < 0.01, and ***p < 0.001 vs. Control.

Previous studies have shown that DS fibroblasts and neural progenitor cells exhibited decreased cell proliferation or increased apoptosis (Kimura et al., 2005; Gimeno et al., 2014; Hibaoui et al., 2014), while DS astrocyte precursor cells exhibited an accelerated proliferation (Kawatani et al., 2021). Since we made the preliminary observation in our experiments that the expansion of DS iPSC lines is slower than control iPSC lines, we wanted to know whether the rate of proliferation or cell death is different in DS from control. We first cultured both DS and control iPSC lines with the same number of live cells (10^5^ cells) at day 0, and total cell numbers were counted at day 3, day 5, and day 7. As shown in Figure 2A, there was a significantly reduced number of DS iPSCs as compared to control lines at 5 and 7 days in culture (Figure 2A and Supplementary Figure 2A, *p* < 0.05 and *p* < 0.001). We next used Ki67, BrdU, and EdU as cell proliferation markers to compare the number of proliferating cells between DS iPSCs and control iPSCs. Ki67 labels cells in the G1, S, G2 and M phase. BrdU and EdU label cells in the S phase only. Interestingly, since most of the DS and control iPS cells were immunoreactive for Ki67^+^ (Figure 2B), we compared and quantified the BrdU^+^ and EdU^+^ cells between the two groups. As shown in Figures 2C,D, DS iPSCs have a significantly decreased number of BrdU^+^ and EdU^+^ cells than control cells (Figures 2C,D and Supplementary Figure 2B, *p* < 0.01), suggesting a decreased proliferation in DS iPSCs. To exclude a potential effect of loss of pluripotency on a decreased proliferation in DS iPSCs, we confirmed that both DS and control iPSCs remain in a stem cell state at day 5, positive for pluripotency markers TRA-1-60, Nanog, and SOX2 and negative for neural progenitor marker nestin (Supplementary Figure 2C). Last, we used cleaved caspase 3 as a cell death marker to compare the number of cell death between DS and control iPSCs. There were no significant differences in cleaved caspase 3^+^ cells between the two groups (Figures 2E,F and Supplementary Figure 2D, *p* = 0.21), suggesting that a slower growth of DS iPSCs is not due to an increased cell death but due to a decreased proliferation.

### Abnormal accumulation of Aß in DS organoids

**Figure 3:**
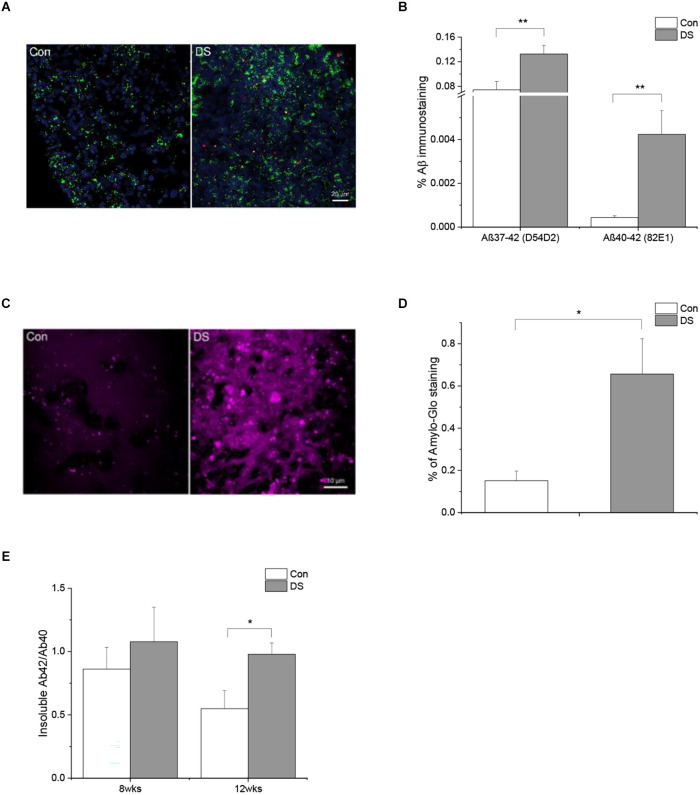
Abnormal Aß accumulation in DS iPSCs-derived cortical organoids. **(A)** A representative Aß immunostaining in 12 weeks DS and isogenic control organoids with two different antibodies D54D2 (Aß37-42, green) and 82E1 (Aß40-42, red). **(B)** Immunohistochemical analysis of Aß antibodies D54D2 and 82E1 revealed a significantly increased Aß immunoreactivity in DS organoids (*n* = 10–11, *p* < 0.01). **(C)** A representative Aß plaque staining in DS and control organoids using Amylo-Glo. **(D)** Immunohistochemical analysis of Amylo-Glo revealed a significantly increased amyloid plaque load in DS organoids (*n* = 8, *p* < 0.05). **(E)** Aß40 and Aß42 in the soluble and insoluble fractions of 8 weeks and 12 weeks DS and control organoids were quantified using Elisa and the ratio of Aß42/Aß40 was significantly increased in the insoluble fractions of 12 weeks DS organoids (*n* = 10–11, *p* < 0.05). *p < 0.05, and **p < 0.01 vs. Control.

Accumulation of Aß is a major AD-like neuropathology feature in DS patients. Aβ is a cleavage product of APP through sequential proteolytic processing by β- and γ-secretases, a process that generates a number of Aβ isoforms with 36–43 amino acid residues in length. Aβ40 and Aβ42 are two major isoforms and the longer isoform, e.g., Aβ42, is more prone to form aggregates and be more toxic. Most amyloid plaques contain beta amyloid with 40 and 42 isoforms (Antzutkin et al., 2000; Balbach et al., 2002; Gu and Guo, 2013). In the current study, we used three different methods to examine the presence of Aß accumulation in DS organoids. First, immunostaining was performed on 8-week-old and 12-week-old organoids using two different Aß antibodies, D54D2 and 82E1 (Horikoshi et al., 2004; Ruiz-Riquelme et al., 2021), that recognize Aß 37–42 and Aß 40–42, respectively. In general, there are much more immunoreactivity of D54D2 than 82E1 in both 8-week and 12- week organoid sections. Immunoreactivity of D54D2 was significantly increased in both 8-week (data not shown) and 12-week-old DS organoids (Figures 3A,B, *p* < 0.01) as compared to control organoids. In contrast, immunoreactivity of 82E1 was hardly detected in 8-week organoid sections but can be detected in the 12-week organoid sections. The difference in 82E1 immunoreactivity between DS and control organoids was significant at 12 weeks (Figures 3A,B and Supplementary Figures 3A,B, *p* < 0.01). Next, we used Amylo-Glo plaque stain reagent to stain the amyloid plaque in the organoid sections and observed a significantly increased Amylo-Glo^+^ staining in DS organoids than control organoids (Figures 3C,D and Supplementary Figure 3C, *p* < 0.05). Lastly, we used Elisa to quantify amyloid beta including Aß40 and Aß42 in the detergent soluble and insoluble fractions of DS and control organoids. No significant difference of Aß42, Aß40, or Aß42/Aß40 was observed in the soluble fraction between the two groups in either 8 weeks or 12 weeks. Instead, we found a significantly increased Aß42/Aß40 in the insoluble fraction of 12 weeks DS organoids than that in control organoids at the same age (Figure 3E and Supplementary Figure 3D, *p* < 0.05).

### Abnormal accumulation of tau pathology in DS organoids

**Figure 4:**
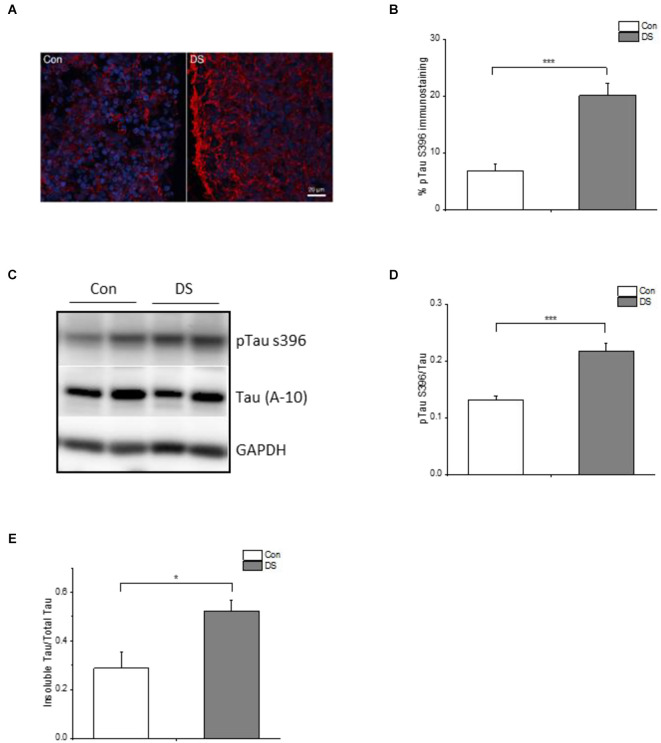
Tau pathology in DS iPSCs-derived cortical organoids. **(A)** A representative pTau S396 (red) and DAPI (blue) immunostaining in 12 weeks DS and isogenic control organoids. **(B)** Immunohistochemical analysis of pTau S396 revealed a significantly increased pTau S396 immunoreactivity in DS organoids (*n* = 24–28, *p* < 0.001). **(C)** A representative Western blot of pTau S396 and Tau expression in 12 weeks DS and isogenic control organoids. **(D)** Immunoblotting analysis of pTau S396 and Tau revealed a significantly increased pTau S396/Tau ratio in DS organoids (*n* = 6, *p* < 0.001). **(E)** Both soluble and insoluble Tau in 12 weeks of DS and control organoids are measured and Elisa data revealed that DS organoids have an increased insoluble Tau/total Tau (soluble Tau + insoluble Tau; *n* = 6, *p* < 0.05). *p < 0.05, and ***p < 0.001 vs. Control.

Hyperphosphorylation of Tau is another AD-like pathological hallmark in DS patients and occurs following Aß accumulation. We, therefore, compared the immunoreactivity of phospho-tau S396 (pTau S396, PHF-1), a widely used antibody to study Tau pathology in AD, in DS and control organoids at 12 weeks (Citron et al., 1994; Foidl and Humpel, 2018; Aragao Gomes et al., 2021). Immunoreactivity of phosphorylated Tau S396 was significantly increased in DS organoids as compared with control organoids (Figures 4A,B and Supplementary Figures 4A,B, *p* < 0.001). This increased phosphorylation of Tau was also confirmed by Western blot when using pTau S396 and total Tau (A-10) antibody: the ratio of pTau S396/Tau was significantly increased in DS organoids than control organoids (Figures 4C,D and Supplementary Figure 4C, *p* < 0.001).

The pathologic hyperphosphorylation of tau proteins induces the formation of insoluble aggregates and neurofibrillary tangles (NFTs) that abnormally accumulate inside neurons in AD or AD-like DS brains (Mondragon-Rodriguez et al., 2014). Levels of soluble and insoluble tau reflect the overall status of tau phosphorylation *in vivo* (Hirata-Fukae et al., 2009) and the insoluble tau correlates with the pathological features of tauopathy (Ren and Sahara, 2013). Therefore, we measured the amount of Tau in the soluble and insoluble fractions of organoids using Elisa. The ratio of insoluble Tau/total Tau was significantly increased in DS organoids at 12 weeks as compared to control organoids (Figure 4E and Supplementary Figure 4D, *p* < 0.05), further confirming a relatively increased insoluble tau aggregates in DS organoids.

## Discussion

Virtually all DS patients over 40 years of age have AD-like neuropathology including abnormal Aß accumulation and neurofibrillary tangles (Wisniewski et al., 1985; Mann and Esiri, 1989). According to the National Down Syndrome Society, about 30% of DS patients are diagnosed with dementia in their 50s, and 50% of DS patients are diagnosed with dementia by their 60s. However, how the disease progresses or how early does it start in DS or AD-DS brain before the clinical onset of the disease is still obscure and much less explored. IPSC-derived 3D organoids closely simulate key endogenous neurodevelopmental events with a cytoarchitecture resembling the developing human brain (Helen Zhao et al., 2021) with the trajectory of human brain development and maturation (Trujillo et al., 2019). Therefore, we used DS-specific iPSC-derived brain organoids as a model system to study the AD-like disease pathology in DS.

Clinically, individuals with DS have an overall reduced brain volume. Hypocellularity has been associated with impaired neurogenesis and lower proliferative rate potency that can be observed as early as 24 weeks during gestation in the DS brain (Stagni et al., 2018; Utagawa et al., 2022). This phenomenon was not only observed in neurons but also in other cell types *in vitro*. For instance, DS fibroblasts have a decreased proliferation (Gimeno et al., 2014); DS iPSC-derived neural progenitor cells show a decreased proliferation and increased apoptosis (Hibaoui et al., 2014); DS iPSC-derived astrocytes have however an increased proliferation rate than control cells (Kawatani et al., 2021). In the current study, we report that DS iPSCs have a slower growth rate and a decreased proliferation than control cells, without any difference in cell death between the two groups.

Abnormal accumulation of extracellular amyloid beta (Aß) and intracellular Tau hyperphosphorylation are two major pathological features in the AD brain and the AD-like DS brain. Aß are by-products of the normal cellular metabolism of amyloid precursor protein (APP; Sinha and Lieberburg, 1999). APP is an integral membrane protein encoded by the APP gene located on chromosome 21. APP has two primary endogenous processing pathways including the non-amyloidogenic pathway and the amyloidogenic pathway. Under physiological conditions, the majority of APP is processed through the non-amyloidogenic pathway; a small portion of APP is processed through the amyloidogenic pathway and yields Aβ 37, Aβ38, Aβ40, and Aβ42. Aβ40 is the most abundant form of Aβ, but Aβ42 is more prone to form insoluble aggregate. Under pathological conditions such as in Alzheimer’s disease and Down syndrome, altered amyloidogenic pathway and increased APP dose promote Aβ accumulation, Aβ aggregation, as well as the formation of insoluble fibrils into amyloid plaques. In DS patients, abnormal Aß accumulation starts as early as 8 years of age (Leverenz and Raskind, 1998). Here *in the organoids*, we were actually able to see increased Aß immunostaining and increased accumulation of amyloid plaque in DS cortical organoids at a very early age in 8 and 12 weeks old organoids in culture. In this regard, it is noteworthy to mention that the difference between both Aß 82E1 immunoreactivity and Aß42/Aß40 was not significant at 8 weeks but started at 12 weeks. Skovronsky previously described that the intracellular pool of insoluble Aß accumulates in a time-dependent manner in NT2N neuronal culture (Skovronsky et al., 1998). A time-dependent increasing difference of Aß between DS and control organoids confirmed that abnormal Aß accumulation is a progressive process and a disease-specific property in DS organoids as compared to controls. These data suggest that disease-specific iPSC-derived organoid culture shed important light on the pathophysiology of DS in that abnormal brain formation starts very early in life, probably in fetal life.

Elevated phosphorylation of Tau and abnormal aggregation are widely considered pathological hallmarks in AD. Here we show that both protein expression and immunoreactivity of pTau S396 are significantly increased in DS organoids, demonstrating hyperphosphorylation of Tau in DS organoids, which in general leads to Tau protein aggregation. Pathological aggregation of Tau protein forms insoluble twisted fibers, named neurofibrillary tangles, inside the cells and acts as a biomarker in AD-like pathology. Consistently, we found an increased composition of insoluble Tau in DS organoids that may therefore contribute to Tau pathology in DS patients. It is worth mentioning that, we have noticed that differences between individual clones (Supplementary Figures) did exist. For example, the differences between two DS lines occurred so that the *p*-value of cleaved caspase 3^+^ cell death did not reach significance (Supplementary Figure 2D, *p* = 0.21). However, differences between DS and their isogenic control in insoluble tau/tau reached significance in spite of differences in DS clones (Supplementary Figure 4D, *p* < 0.02). We speculate that the differences between individual lines might be a consequence of differentiation efficiency among batches, but that the difference observed between DS and control is due to disease phenotype. In the current study, we used isogenic lines to avoid genetic variations and confounding factors.

Recent Tau studies, however, lead us to reconsider the role of Tau phosphorylation in Alzheimer’s disease (Wegmann et al., 2021). Due to the fact that the phosphorylation patterns of physiological and pathological Tau are surprisingly similar and heterogenous, and the phosphorylation levels of Tau seem insufficient to differentiate between healthy and diseased Tau, high phosphorylation does not necessarily lead to Tau aggregation (Wegmann et al., 2021). In addition, the posttranslational modification of Tau other than phosphorylation such as ubiquitination, acetylation, and methylation may also regulate the formation of Tau aggregates (Wegmann et al., 2021). Indeed, abnormal posttranslational modifications have been reported in DS patients (Kerkel et al., 2010; Jones et al., 2013; Sailani et al., 2015; Tramutola et al., 2017) and may need additional examination in future studies.

In contrast to a previously published article on DS organoids (Gonzalez et al., 2018), we reported a difference between the DS iPSC lines and their isogenic controls at the iPSC stage. Furthermore, we systemically quantify the abnormalities of AD-like neuropathological phenotype at two time points and demonstrated that the AD-like neuropathology progressively manifests itself in organoids during early development. In summary, we have demonstrated that DS disease-specific iPS cells have a slower growth rate due to decreased proliferation. DS iPSC-derived brain organoids mimic AD-like pathophysiological phenotype including abnormal Aß accumulation and insoluble Tau accumulation. Our data strongly suggest that DS iPSC-derived cortical organoids illustrate well the notion that the molecular pathobiology in DS starts early in brain development and can be used as a model system to study the AD-like pathology and its progress before the clinical onset of the disease.

## Data Availability Statement

The raw data supporting the conclusions of this article will be made available by the authors, without undue reservation.

## Ethics Statement

The studies involving human participants were reviewed and approved by IRB at the University of California San Diego. Written informed consent for participation was not required for this study in accordance with the national legislation and the institutional requirements.

## Author Contributions

HZ and GH designed the experiments and wrote the manuscript. HZ performed the experiments and data analysis. All authors contributed to the article and approved the submitted version.

## Supplementary Material

Supplementary Figure 1 Corresponding to Figure 1B: expression profile of chromosome 21 genes from individual DS iPSC and isogenic control iPSC lines.

Supplementary Figure 2 **(A)** Corresponding to Figure 2A: total cell numbers were counted after 3, 5, and 7 days in culture from individual DS iPSC and isogenic control iPSC lines.** (B)** Corresponding to Figure 2D: the number of BrdU^+^ and EdU^+^ cells was summarized from individual DS iPSC and isogenic control iPSC lines. **(C)** A representative double labeling of SOX2 (red) and Tra-1-60 (green) in DS and control iPSCs at day 5 (top panel); a representative double labeling of Nestin (red) and Nanog (green) in DS and control iPSCs at day 5 (bottom panel left) and in 4-week old control organoids (bottom panel right). Immunostaining of organoids was used as a positive control for Nestin (red) antibody under the same conditions of immunostaining and imaging processes. **(D)** Corresponding to Figure 2F: the number of cleaved caspase 3^+^ cells was summarized from individual DS iPSC and isogenic control iPSC lines.

Supplementary Figure 3 **(A)** Corresponding to Figure 3B: immunoreactivity of Aß antibody D54D2 and 82E1 was summarized from individual DS and isogenic control iPSC line derived organoids respectively.** (B)** A representative double labeling of Aß antibody 82E1 (red) and MAP2 (green) in DS and control organoids (top panel). A representative double labeling of S100 (red) and MAP2 (green) in DS and control organoids (bottom panel). **(C)** Corresponding to Figure 3D: immunoreactivity of Amylo-Glo was summarized from individual DS and isogenic control iPSC line derived organoids. **(D)** Corresponding to Figure 3E: ratio of Aß42/Aß40 was summarized from individual DS and isogenic control iPSC line derived organoids.

Supplementary Figure 4 **(A)** Corresponding to Figure 4B: immunoreactivity of pTau S396 was summarized from individual DS and isogenic control iPSC line derived organoids. **(B)** A representative double labeling of pTau S396 (red) and MAP2 (green) in DS and control organoids. **(C)** Corresponding to Figure 4E: immunoblotting analysis of pTau S396/Tau was summarized from individual DS and isogenic control iPSC line derived organoids. **(D)** Corresponding to Figure 4D: the ratio of insoluble Tau/total Tau (soluble Tau + insoluble Tau) was summarized from individual DS and isogenic control iPSC line derived organoids.
