# Supplementary figures and images for: Alzheimer’s disease like neuropathology in Down syndrome cortical organoids
Source: Front Cell Neurosci. 2022 Dec 8;16:1050432. doi: 10.3389/fncel.2022.1050432 (PMC9773144; doi:10.3389/fncel.2022.1050432)

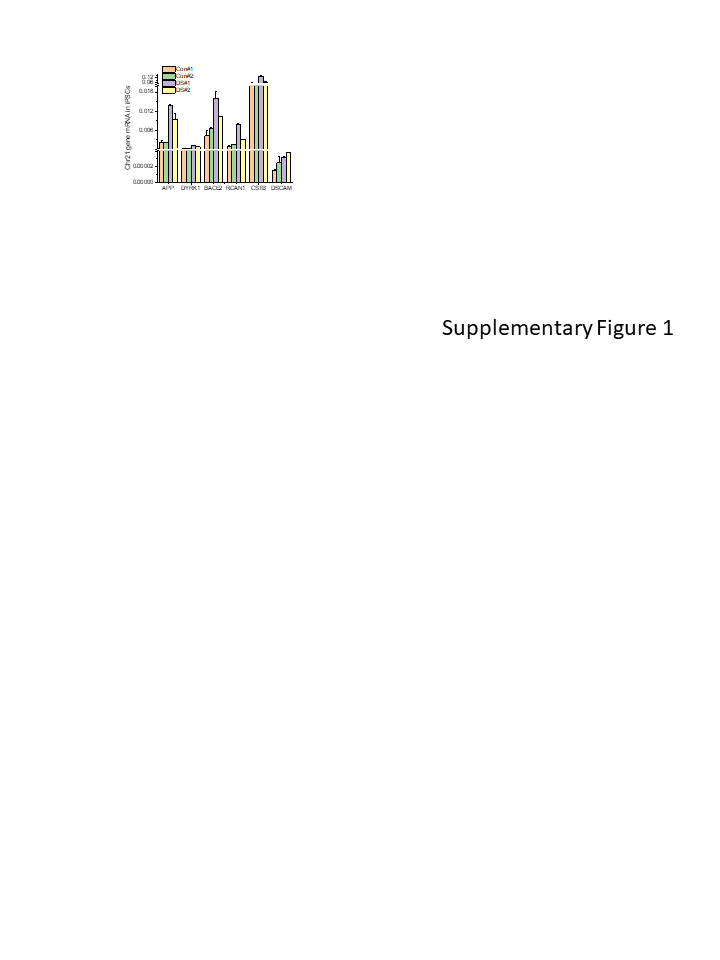

Supplement: Supplementary Figure 1 — Corresponding to Figure 1B: expression profile of chromosome 21 genes from individual DS iPSC and isogenic control iPSC lines. [file Image_1.tif]

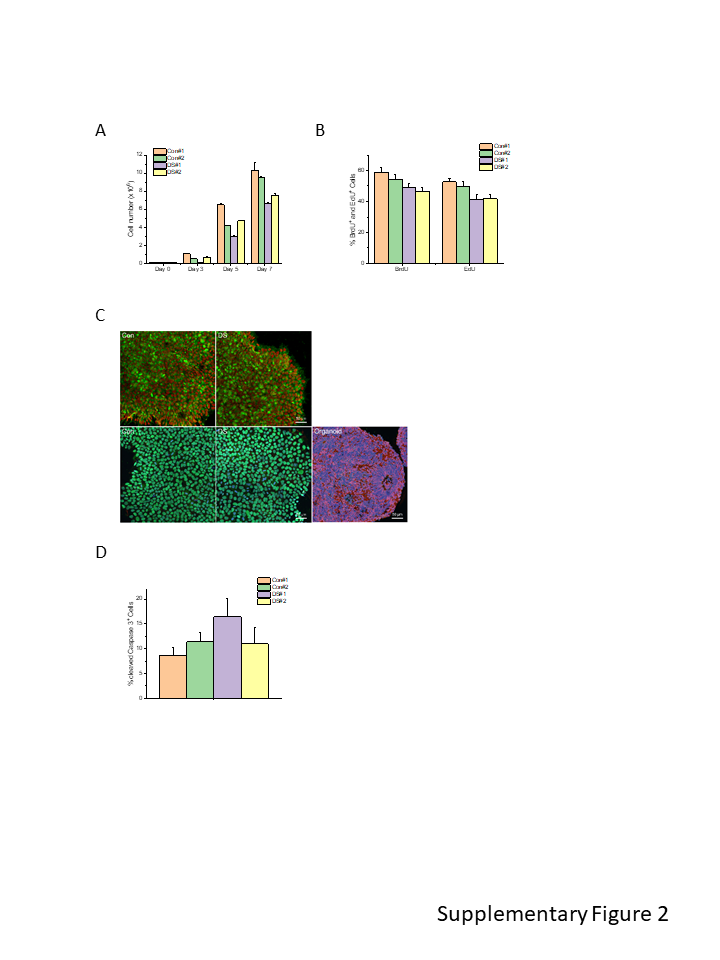

Supplement: Supplementary Figure 2 — (A) Corresponding to Figure 2A: total cell numbers were counted after 3, 5, and 7 days in culture from individual DS iPSC and isogenic control iPSC lines. (B) Corresponding to Figure 2D: the number of BrdU+ and EdU+ cells was summarized from individual DS iPSC and isogenic control iPSC lines. (C) A representative double labeling of SOX2 (red) and Tra-1-60 (green) in DS and control iPSCs at day 5 (top panel); a representative double labeling of Nestin (red) and Nanog (green) in DS and control iPSCs at day 5 (bottom panel left) and in 4-week old control organoids (bottom panel right). Immunostaining of organoids was used as a positive control for Nestin (red) antibody under the same conditions of immunostaining and imaging processes. (D) Corresponding to Figure 2F: the number of cleaved caspase 3+ cells was summarized from individual DS iPSC and isogenic control iPSC lines. [file Image_2.tif]

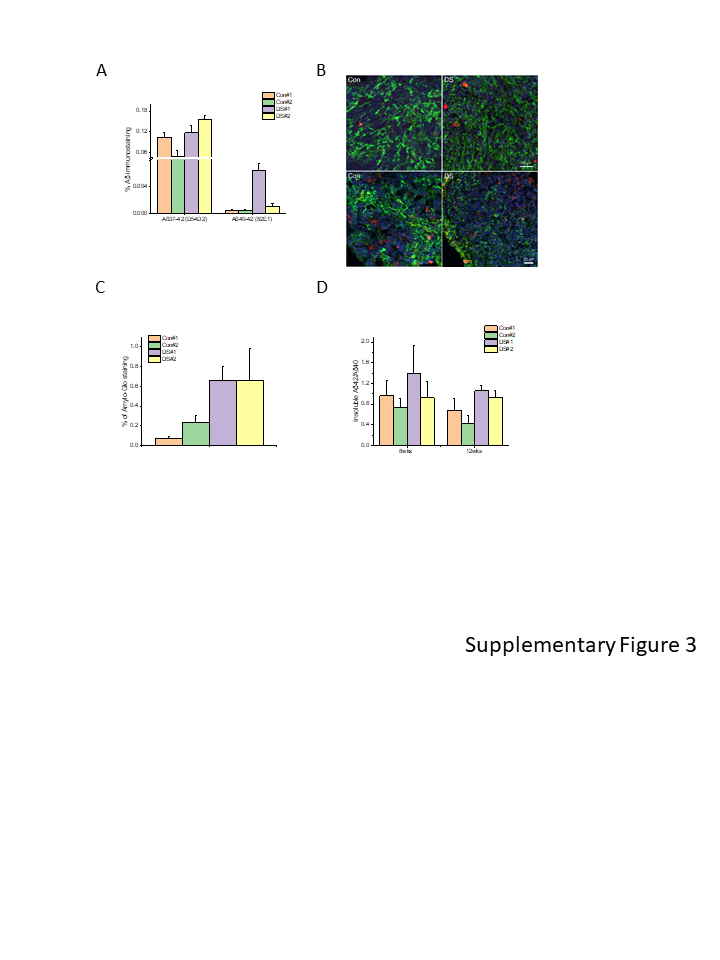

Supplement: Supplementary Figure 3 — (A) Corresponding to Figure 3B: immunoreactivity of Aß antibody D54D2 and 82E1 was summarized from individual DS and isogenic control iPSC line derived organoids respectively. (B) A representative double labeling of Aß antibody 82E1 (red) and MAP2 (green) in DS and control organoids (top panel). A representative double labeling of S100 (red) and MAP2 (green) in DS and control organoids (bottom panel). (C) Corresponding to Figure 3D: immunoreactivity of Amylo-Glo was summarized from individual DS and isogenic control iPSC line derived organoids. (D) Corresponding to Figure 3E: ratio of Aß42/Aß40 was summarized from individual DS and isogenic control iPSC line derived organoids. [file Image_3.tif]

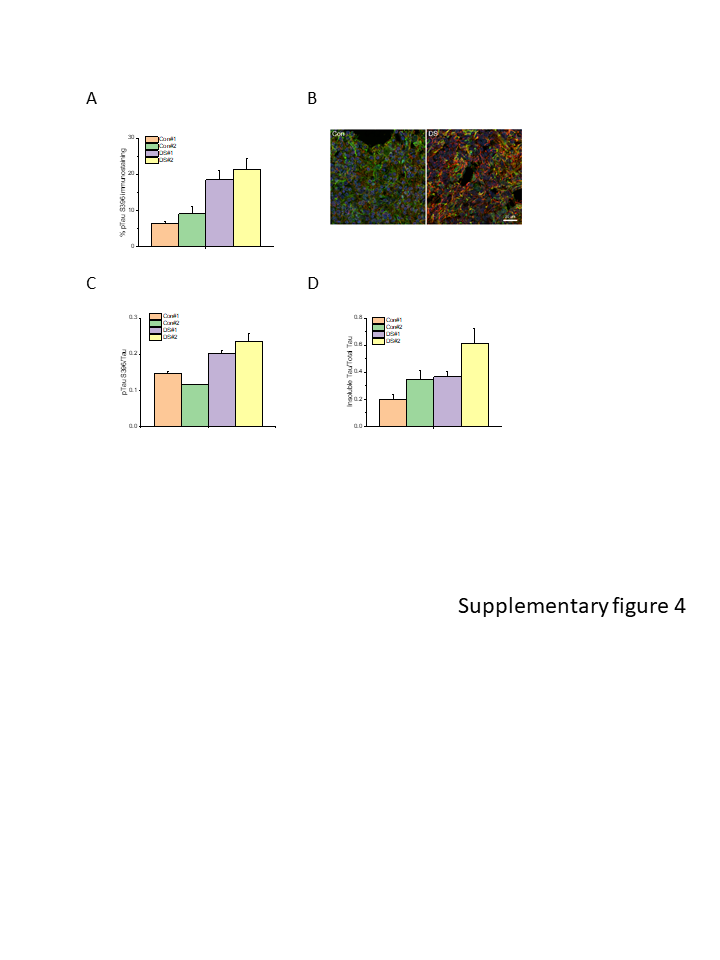

Supplement: Supplementary Figure 4 — (A) Corresponding to Figure 4B: immunoreactivity of pTau S396 was summarized from individual DS and isogenic control iPSC line derived organoids. (B) A representative double labeling of pTau S396 (red) and MAP2 (green) in DS and control organoids. (C) Corresponding to Figure 4E: immunoblotting analysis of pTau S396/Tau was summarized from individual DS and isogenic control iPSC line derived organoids. (D) Corresponding to Figure 4D: the ratio of insoluble Tau/total Tau (soluble Tau + insoluble Tau) was summarized from individual DS and isogenic control iPSC line derived organoids. [file Image_4.tif]
